# Investigating the NPY/AgRP/GABA to GnRH Neuron Circuit in Prenatally Androgenized PCOS-Like Mice

**DOI:** 10.1210/jendso/bvaa129

**Published:** 2020-08-31

**Authors:** Christopher J Marshall, Melanie Prescott, Rebecca E Campbell

**Affiliations:** Centre for Neuroendocrinology and Department of Physiology, School of Biomedical Sciences, University of Otago, Dunedin, New Zealand

**Keywords:** neuropeptide Y, steroid hormone receptors, transgenic mouse, tract-tracing, HPG axis, hypothalamic-pituitary-gonadal axis

## Abstract

Polycystic ovary syndrome (PCOS), the most common form of anovulatory infertility, is associated with altered signaling within the hormone-sensitive neuronal network that regulates gonadotropin-releasing hormone (GnRH) neurons, leading to a pathological increase in GnRH secretion. Circuit remodeling is evident between GABAergic neurons in the arcuate nucleus (ARN) and GnRH neurons in a murine model of PCOS. One-third of ARN GABA neurons co-express neuropeptide Y (NPY), which has a known yet complex role in regulating GnRH neurons and reproductive function. Here, we investigated whether the NPY-expressing subpopulation (NPY^ARN^) of ARN GABA neurons (GABA^ARN^) is also affected in prenatally androgenized (PNA) PCOS-like NPY^ARN^ reporter mice [Agouti-related protein (AgRP)-Cre;τGFP]. PCOS-like mice and controls were generated by exposure to di-hydrotestosterone or vehicle (VEH) in late gestation. τGFP-expressing NPY^ARN^ neuron fiber appositions with GnRH neurons and gonadal steroid hormone receptor expression in τGFP-expressing NPY^ARN^ neurons were assessed using confocal microscopy. Although GnRH neurons received abundant close contacts from τGFP-expressing NPY^ARN^ neuron fibers, the number and density of putative inputs was not affected by prenatal androgen excess. NPY^ARN^ neurons did not co-express progesterone receptor or estrogen receptor α in either PNA or VEH mice. However, the proportion of NPY^ARN^ neurons co-expressing the androgen receptor was significantly elevated in PNA mice. Therefore, NPY^ARN^ neurons are not remodeled by prenatal androgen excess like the wider GABA^ARN^ population, indicating GABA-to-GnRH neuron circuit remodeling occurs in a presently unidentified non-NPY/AgRP population of GABA^ARN^ neurons. NPY^ARN^ neurons do, however, show independent changes in the form of elevated androgen sensitivity.

Polycystic ovary syndrome (PCOS) is the most common form of anovulatory infertility, affecting 8% to 10% of women of reproductive age [1]. While it is typically described as a highly heterogeneous disorder, neuroendocrine impairment is a consistent feature among women with PCOS. From 50% to 75% of women with PCOS exhibit evidence of luteinizing hormone (LH) hypersecretion [2-4], and 90% have an elevated ratio of LH to follicle-stimulating hormone secretion [4]. Serial blood sampling shows that this hypersecretion reflects a persistently elevated LH pulse frequency [5, 6], indicating a net increase in the activity of the gonadotropin-releasing hormone (GnRH) neural network. This elevated hypothalamic output is likely to stem in part from diminished negative feedback by ovarian steroid hormones, as exogenous estradiol and progesterone are less effective at reducing LH secretion in women with PCOS [7, 8]. Androgen excess, a key feature of PCOS, may impede negative feedback, as long-term blockade of the androgen receptor in women with PCOS can restore sensitivity to ovarian steroid hormones [9]. Such observations have spurred research in animal models using androgen excess to recapitulate PCOS-like features [10] to determine the loci of disrupted hormone sensitivity and associated circuit alterations within the GnRH neural network.

Functional and anatomical findings in both ovine and murine models of PCOS suggest that altered afferent GABAergic input to GnRH neurons may play a role in elevated GnRH/LH secretion [11-19]. Prenatally androgenized (PNA) mice, which reflect the cardinal features and neuroendocrine impairments of PCOS [20], display a greater frequency of GABAergic postsynaptic currents in GnRH neurons [13, 15] and elevated GnRH neuron firing frequency [15, 19, 21] compared with fertile controls. This is associated with a greater number of closely associated presynaptic GABAergic terminals and a dramatically increased projection of GABAergic fibers from the arcuate nucleus (ARN) [14]. Elevated GABAergic input to GnRH neurons appears to be due to early network organization, as both presynaptic markers for GABAergic terminals and postsynaptic GABA currents are elevated by 3 weeks of age [16, 22]. The ARN GABA neuron population (GABA^ARN^) in PNA mice also exhibits a reduction in progesterone receptor expression, indicating reduced sensitivity to this important feedback cue [14]. Specific activation of the GABA^ARN^-to-GnRH neuron circuit with optogenetic and chemogenetic tools elicits LH secretion and mimics some features of PCOS, such as disrupted ovulatory cycles, reduced presence of corpora lutea in the ovary, and an increase in circulating testosterone [17], suggesting that modifications in this circuit may underpin the elevated LH secretion evident in PNA-treated, PCOS-like mice.

GABA^ARN^ neurons in rodents are a large heterogeneous population of neurons that co-secrete a range of neuropeptides and transmitters with implicated roles in the control of GnRH neurons [23-31]. What has remained unclear is whether PNA-induced circuit alternations occur in GABA^ARN^ neurons as a whole, or within particular subsets of the population. Neuropeptide Y (NPY) is co-expressed in one-third of the GABA^ARN^ population in both fertile and PNA-treated female mice, and nearly all NPY neurons in the ARN are GABAergic [31]. NPY, along with co-expressed agouti-related protein (AgRP), is a well-established regulator of energy homeostasis [32] that is also highly implicated in the regulation of the reproductive axis [33-36]. NPY can exert direct effects upon GnRH neurons [37, 38], while selective activation of ARN NPY/AgRP neurons regulates upstream kisspeptin neurons via both GABA [39] and NPY receptor-dependent mechanisms [36]. Additionally, selective activation of ARN NPY (NPY^ARN^) neurons can cause potent modulation of GnRH/LH secretion [35]. Anatomical evidence suggests that NPY^ARN^ neurons innervate the proximal region of GnRH neurons [40] and that synapses formed by NPY^ARN^ neurons in these proximal regions are GABAergic in nature [40, 41]. Given that elevated GABA^ARN^ input is largely to the proximal dendrite of GnRH neurons in PCOS-like animals [12, 14] and the high degree of NPY co-expression in GABA^ARN^ neurons [31], we aimed to investigate whether the NPY/AgRP-specific subpopulation of GABA^ARN^ neurons is remodeled in the PNA mouse model of PCOS. This was achieved using transgenic AgRP-Cre;τGFP reporter mice to specifically visualize NPY^ARN^ cell bodies and fiber projections. We hypothesized that the NPY^ARN^-to-GnRH neuron circuit and the steroid hormone sensitivity of NPY^ARN^ neurons would be impacted in PNA-induced PCOS-like mice.

## 1. Methods

### Animals and tissue collection

The following procedures were carried out with permission from the University of Otago Animal Ethics Committee (Dunedin, New Zealand). Adult female mice were generated and housed in the University of Otago Biomedical Research Facility. Mice were kept in individually ventilated cages, in a climate-controled environment (20 °C, 40% humidity) on a 12:12 hour light:dark cycle. Mice were provided *ad libitum* access to food and water.

Mice in which ARN NPY neurons were identified by green fluorescent protein (GFP) were generated by crossing AgRP-IRES-Cre mice [42] with ROSA26-CAGS-τGFP floxed-stop reporter mice [43] to generate AgRP-Cre;τGFP mice. Prenatal androgen excess treatment was performed as described previously [13, 20], by injection of dams on days 16, 17, and 18 of pregnancy with 100 µL of sesame oil alone (vehicle controls, VEH) or containing 250 µg of di-hydrotestosterone (DHT) (prenatally androgenized, PNA). Induction of a PCOS-like phenotype was assessed by daily vaginal cytology smears for 14 days to ensure PNA-treated mice were acyclic (Supplemental Fig. 1 [49]). Female offspring were studied in adulthood (60-90 days) during diestrus, assessed by vaginal cytology. Following a lethal dose of pentobarbital (3 mg/mL, 100 µL intraperitoneal), animals underwent transcardial perfusion with 4% paraformaldehyde to fix the brain. The brain was then dissected from the skull, cryoprotected in 30% sucrose, and cut on a freezing microtome into 30-µm thick coronal sections.

### Experiment 1: assessing NPY^ARN^ neuron projections to GnRH neurons

#### Double-label fluorescent immunohistochemistry.

 To assess NPY^ARN^-to-GnRH neuron circuitry, free-floating immunohistochemistry was performed on every third coronal section through the rostral forebrain including the medial septum (MS; bregma +1.34 to +0.74 mm), rostral preoptic area (rPOA; bregma +0.74 to +0.38 mm) and anterior hypothalamic area (AHA; bregma +0.38 to -0.46 mm) populations of GnRH neurons from VEH (n = 5) and PNA (n = 8) mice. GnRH neurons were labeled using a guinea pig anti-GnRH primary antibody (1:5000; GA2, kindly gifted by Prof Greg Anderson; RRID:AB_2721118) [44] and NPY^ARN^ neuron fibers were labeled for the τGFP reporter using a chicken anti-GFP primary antibody (1:5000; Aves Labs, OR, USA; RRID:AB_10000240) [45]. GFP-positive fibers were amplified using a goat anti-chicken AlexaFluor488 antibody (1:200; Molecular Probes, OR, USA), while GnRH neurons were visualized using a goat anti-guinea pig AlexaFluor568 antibody (1:200; Molecular Probes, OR, USA). Specificity of secondary antibodies was assessed by primary antibody omission in negative control tissue sets. Mounted sections were coverslipped with Fluoromount G (ThermoFisher Scientific, MA, USA) and kept in the dark at 4 ºC until imaging.

#### Image acquisition and analysis.

 Confocal microscopy was performed using a Nikon A1R multi-photon microscope (Nikon Instruments Inc., Melville, NY, USA) to collect images of individual GnRH neurons in the MS, rPOA, and AHA. As reported previously [14, 16, 18], in each animal, z-stack confocal images were collected from 5 GnRH neurons in the MS and AHA, and 10 GnRH neurons in the rPOA, reflecting their distribution density. Using a Plan-Neofluar 40X oil objective (1.30 NA) and 3× digital zoom, scans throughout the soma and the first 75 µm of the primary dendrite of each GnRH neuron were performed using 0.5 µm z-intervals. Pinhole size was maintained at 1 AU using a consistent laser power across animals, while digital gain and offset of red and green channels was kept consistent (<5% variation) to prevent imaging artifacts from confounding or biasing later analysis.

Images of individual GnRH neurons and surrounding GFP-expressing NPY^ARN^ neuron fibers were analyzed using NIS Elements software (Nikon Instruments Inc.). The soma circumference of each neuron was measured using pre-established pixel-to-µm conversion preprogramed into the software; each soma was measured using the image in the stack where the soma was at its largest, and this measurement was recorded. Each primary dendrite was divided into 15-µm segments up to 75 µm, using the measurement tool and manual demarcation of each segment. The number of GFP-expressing fiber contacts associated with each GnRH neuron was recorded, along with the location of contact (soma or individual dendrite segments). A contact was defined as the point where the red GnRH and green GFP label were contiguous without intervening black pixels, and required that this was present in both the XY plane of view and the orthogonal YZ view of a single focal plane. When a fiber passed across the soma or a segment of dendrite, or “bundled” with the neuron before projecting away, this was recorded as one point of contact. These data were used to calculate the total number of contacts at the level of the soma, primary dendrite as a whole, and within 15-µm segments of the dendrite. To calculate the density of appositions, the total number of contacts at the soma were divided by the soma circumference, and the number of contacts within each segment of dendrite were divided by 15, giving a density in contacts/µm. This provided a measurement of the putative innervation density to each GnRH neuron.

### Experiment 2: assessing steroid hormone receptor expression in NPY^ARN^ neurons

#### Double-label fluorescent immunohistochemistry.

To investigate NPY^ARN^ co-expression with gonadal steroid hormone receptors, free-floating immunohistochemistry was performed on coronal sections throughout the rostral, middle, and caudal ARN (rARN, mARN, cARN). NPY^ARN^ neuron somata were labeled for GFP reporter expression using a chicken anti-GFP primary antibody (1:5000; Aves Labs) [45] as above. Every third section was co-labeled for one of the following steroid hormone receptors: progesterone receptor (rabbit anti-PR, 1:100; Dako (Agilent), CA, USA; VEH n = 8, PNA n = 10; RRID:AB_2315192) [46], estrogen receptor α (rabbit anti-ERα, 1:5000; Millipore; VEH n = 5, PNA n = 5, RRID:AB_310305) [47], or androgen receptor (rabbit anti-AR PG-21, 1:200; Millipore, MA, USA; VEH n = 4, PNA n = 4; RRID:AB_310214) [48]. To amplify GFP signal, a goat anti-chicken AlexaFluor488 antibody was used (1:200; Molecular Probes, OR, USA), while steroid hormone receptors were labeled using a goat anti-rabbit AlexaFluor568 antibody (1:200; Molecular Probes, OR, USA).

#### Image acquisition and analysis.

Confocal microscopy was performed using a Zeiss LSM710 upright microscope (Carl Zeiss AG, Oberkochen, Germany). Confocal z-stacks of 2 representative sections of the rARN, mARN, and cARN were collected from each animal in each experiment using a PlanApo 20x air objective (0.80 NA) to capture one hemisphere in the visual field, using a 2.12 µm z-interval with a pinhole size of 1 AU. High power images for illustrative purposes were collected using a PlanApo 40× oil objective (1.30 NA) with 2× digital zoom to resolve individual soma, using a 0.5 µm z-interval with a pinhole size of 1 AU.

Images were analyzed using ImageJ software (National Institutes of Health, Bethesda, MD, USA). The number of cell bodies expressing GFP in each 20× image within each respective stack (z-depth 15-25 µm) was counted, along with the number of cells positive for either PR, ERα, or AR, and finally, the number of double-labeled cells in the visual field (a unilateral hemisphere of the ARN). Using this, the percentage of GFP-positive NPY^ARN^ neurons co-labeled with each receptor was calculated.

### Statistical analysis

Statistical analysis was performed using PRISM software (Graphpad Software Inc., LA Jolla, CA, USA). Normality of data was assessed by Shapiro-Wilk tests prior to statistical comparisons between VEH- and PNA-treatment groups. Where values from the entire ARN were grouped, VEH and PNA group means were compared using 2-tailed unpaired Student *t* tests to compare absolute values, or Mann-Whitney U tests to compare percentage means. Where VEH and PNA group means were compared in the rARN, mARN, and cARN separately, a 2-way analysis of variance (ANOVA) was used, with post hoc analysis performed using Bonferroni multiple comparisons tests. Mean number and density of appositions onto GnRH neurons were compared between VEH and PNA groups using a 1-way ANOVA, and post hoc analysis was performed using Tukey’s multiple comparisons tests. *P*-values < 0.05 were considered statistically significant.

## 2. Results

### NPY^ARN^ projections to GnRH neurons are unaffected in PNA mice

Vaginal cytology, collected daily for 2 weeks prior to tissue collection, demonstrated that all PNA mice were acyclic, spending the majority of time in persistent diestrus and never exhibiting a proestrus smear (Supplemental Fig. 1[49]) as expected. In contrast, all VEH control mice cycled normally, completing 1.6 ± 0.2 full estrous cycles (quantified as proestrus day to proestrus day), and spending 18.8 ± 1.3% of the time in proestrus.

**Figure 1.**
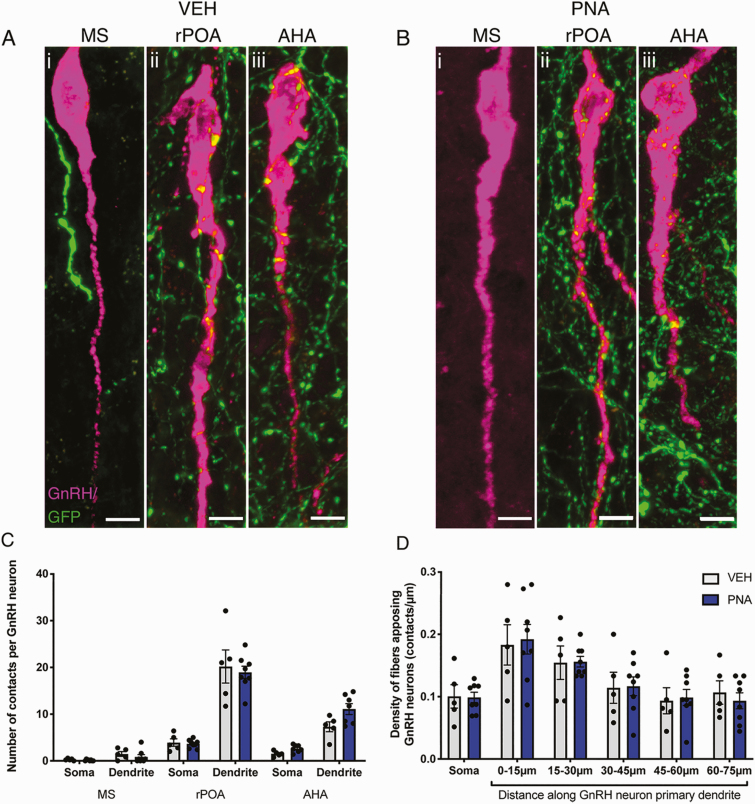
**Presence of close appositions between NPY** ^**ARN**^  **neurons and GnRH neurons is region-dependent and not modified by PNA treatment.** (A,B) Representative images composed from z-projections (thickness 12-14 µm) of GnRH neurons (magenta) residing in the MS (i), rPOA (ii) and AHA (iii) of VEH- (A) and PNA-treated (B) mice showing GFP-expressing NPY^ARN^ fibers (green) in close contact with GnRH neurons. (C) The mean number of contacts per GnRH neuron made by NPY^ARN^ neuron fibers to GnRH neurons located in the MS, rPOA, and AHA, at the level of the soma and the primary dendrite in VEH- (gray bars, n = 5) and PNA-treated mice (blue bars, n = 8). (D) The mean density of contacts made onto GnRH neurons in the rPOA by NPY^ARN^ fiber projections, at the level of the soma and in 15-µm subsections of the primary dendrite. Results are presented as mean ± SEM. No significant differences were detected by 2-way ANOVA (C) or 1-way ANOVA (D). Scale bars = 5 µm.

Dense collections of GFP-immunoreactive (-ir) NPY^ARN^ neuron fibers were observed in close proximity to GnRH neurons located in the rPOA and AHA of both groups (Fig. 1Aii-iii and 1Bii-iii). In contrast, NPY^ARN^ neuron fibers were not as abundant in the MS of either group (Fig. 1Ai and 1Bi). Of the GnRH neurons imaged across both groups, 117/120 in the rPOA and 56/60 neurons in the AHA received at least one close apposition from a GFP-ir fiber, while just 3/60 neurons in the MS received any close appositions.

The mean number of close appositions per GnRH neuron in each region, compared by 2-way ANOVA, was not different between VEH- and PNA-treated groups (F [1, 66] = 0.32, *P* = 0.57; Fig. 1C). Likewise, the mean density of contacts to GnRH neuron somata and primary dendrites in the rPOA, compared using a 1-way ANOVA, was not different between between VEH and PNA mice (F [1, 66] = 0.027, *P* = 0.87; Fig. 1D).

### Assessing steroid hormone receptor expression in NPY^ARN^ neurons in PNA mice

#### Androgen receptor.

**Figure 2.**
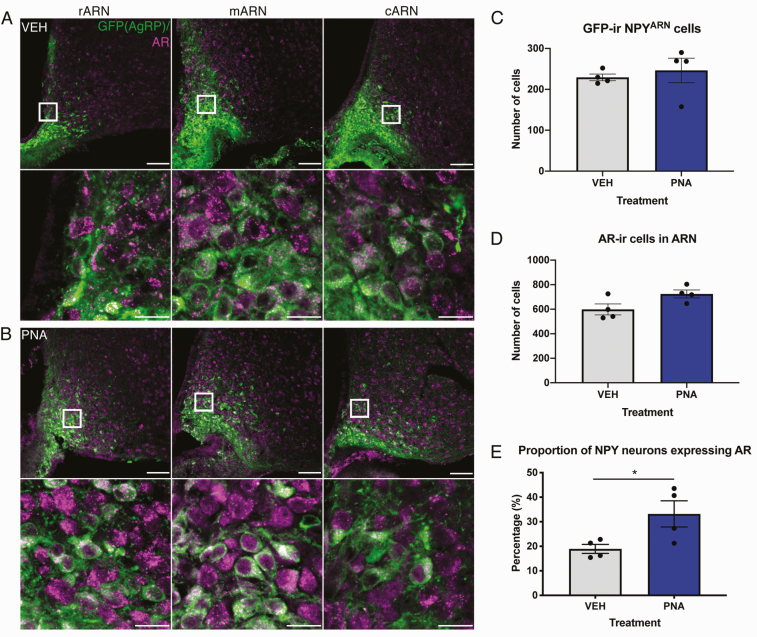
**Expression of AR within NPY** ^**ARN**^  **neurons is elevated in PNA-treated mice.** (A, top) Representative images composed from z-projections (6.36-µm thickness) collected throughout the ARN of a VEH-treated mouse showing AR-ir (magenta) and τGFP reporter expression of NPY^ARN^ neurons (green). (A, bottom) single plane high magnification images taken within the highlighted area indicated in the top row where GFP and AR labeling is observed. (B, top) Representative images composed from 6.36 µm thick z-projections containing AR and GFP-ir in the ARN of a PNA-treated mouse. (B, bottom) single plane high magnification images taken within the highlighted area. (C) Mean number of GFP-expressing NPY neurons counted in VEH- (gray bars, n = 4) and PNA-treated mice (blue bars, n = 4) across the whole ARN. (D) Mean number of AR-ir cells across the entire ARN in VEH- and PNA-treated mice. (D) The percentage of NPY neurons co-expressing AR in VEH- and PNA-treated mice, averaged across the whole ARN. Results are presented as mean ± SEM. * *P* < 0.05 as determined by Mann-Whitney U test. Scale bars = 100 µm (top), 10 µm (bottom).

 Androgen receptor (AR)-ir was evident in the nuclei and cytoplasm of neurons scattered throughout the ARN of both VEH- and PNA-treated female mice (Fig. 2A and 2B), including within the ventromedial subregions where NPY neurons reside. High-magnification images revealed that AR-ir co-localized within NPY neurons, where it was observed as low-level labeling in the nucleus as well as bright puncta aggregated within the cytoplasm (Fig. 2A and 2B). The number of GFP-expressing NPY neurons was not different between in the ARN of VEH- and PNA-treated mice (VEH 229.3 ± 8.18 cells vs PNA 246.4 ± 30.04 cells; Fig. 2C, Table 1). Although total numbers of AR-ir cells in the whole ARN were not significantly different between VEH- and PNA-treated mice (VEH 598.4 ± 44.79 cells vs PNA 724.4 ± 32.35 cells; *P* = 0.067; Fig. 2D), a greater proportion of NPY^ARN^ neurons were identified to co-express AR in PNA-treated mice (33.2 ± 5.3%) compared with VEH-treated mice (18.9 ± 1.8%; *P* = 0.045; Fig. 2E). No significant differences in AR-ir co-expression were identified in specific ARN subregions (Table 1); however, the number of AR-ir cells in the rostral subdivision of the ARN was significantly elevated in PNA mice (Table 1).

**Table 1.** The Mean Number of Steroid Hormone Receptor-Positive and GFP-Positive Cells in the Rostral, Middle, and Caudal ARN of VEH- and PNA-Treated Mice

	rARN			mARN			cARN		
	AR	GFP	%	AR	GFP	%	AR	GFP	%
**VEH**	222.6 ± 16.6	82.0 ± 5.9	22.7 ± 1.6 %	237.8 ± 23.6	88.1 ± 4.0	22.3 ± 3.4 %	138.0 ± 9.27	59.1 ± 5.7	15.2 ± 2.8
**PNA**	300.5 ± 7.6 **	109.9 ± 16.7	32.6 ± 6.1 %	245.4 ± 6.9	86.4 ± 6.0	32.6 ± 5.1 %	178.5 ± 21.1	50.1 ± 14.2	34.4 ± 5.4 %
***P* value**	**0.007**	**0.19**	**0.62**	**>0.99**	**>0.99**	**0.57**	**0.25**	**>0.99**	**0.06**
	**ERα**	**GFP**	**%**	**ERα**	**GFP**	**%**	**ERα**	**GFP**	**%**
**VEH**	148.7 ± 7.2	92.8 ± 6.9	0.0 ± 0.0 %	205.6 ± 8.3	103.2 ± 7.5	0.0 ± 0.0 %	159.4 ± 7.3	93.7 ± 7.0	0.0 ± 0.0 %
**PNA**	180.9 ± 9.6	101.5 ± 8.7	0.12 ± 0.06 %	210.7 ± 12.1	111.4 ± 9.0	0.19 ± 0.10 %	178.1 ± 11.8	104.0 ± 8.9	0.13 ± 0.07 %
***P* value**	**0.08**	**>0.99**	**0.40**	**>0.99**	**>0.99**	**0.07**	**0.54**	**>0.99**	**0.32**
	**PR**	**GFP**	**%**	**PR**	**GFP**	**%**	**PR**	**GFP**	**%**
**VEH**	123.9 ± 4.9	89.6 ± 9.0	0.46 ± 0.21 %	153.7 ± 9.0	105.5 ± 11.9	0.31 ± 0.16 %	134.0 ± 12.5	91.8 ± 9.5	0.36 ± 0.22 %
**PNA**	87.0 ± 8.2 *	95.9 ± 9.1	0.35 ± 0.16 %	121.3 ± 7.6	120.8 ± 13.9	0.24 ± 0.08 %	116.9 ± 12.8	100.1 ± 11.8	0.17 ± 0.07 %
***P* value**	**0.03**	**>0.99**	**>0.99**	**0.07**	**>0.99**	**>0.99**	**0.66**	**>0.99**	**>0.99**

Results are presented as mean ± SEM. Columns with % report the proportion of GFP-expressing cells co-localized with steroid hormone receptors.

Abbreviations: AR, androgen receptor; ARN, arcuate nucleus; cARN, caudal arcuate nucleus; ERα, estrogen receptor alpha; GFP, green fluorescent protein; mARN, middle arcuate nucleus; PNA, prenatally androgenized; PR, progesterone receptor; rARN, rostral arcuate nucleus; VEH, vehicle control.

* *P* < 0.05, ** *P* < 0.01, VEH vs PNA within ARN region.

#### Progesterone receptor.

**Figure 3.**
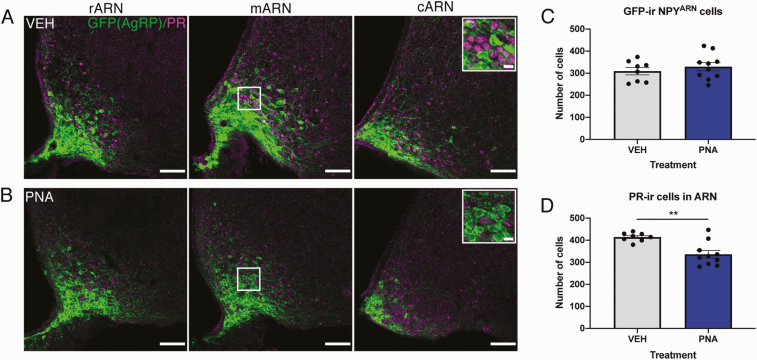
**Fewer PR-ir cells in the ARN of PNA mice and near complete absence of PR in NPY neurons.** (A) Representative single z-plane images from rostral to caudal zones in the ARN showing PR-ir (magenta) and amplified τGFP reporter expression of NPY^ARN^ neurons (green) in a VEH-treated mouse (A) and a PNA-treated mouse (B). Inset boxes show enlarged regions of the middle ARN indicating that although PR-ir cells and τGFP expressing NPY^ARN^ neurons are in close proximity, there is an almost complete absence of co-localization. (C) Mean number of GFP-positive NPY neurons across the whole ARN in VEH- (gray bars, n = 8) and PNA-treated (blue bars, n = 10) mice. (D) Mean number of PR-ir cells across the whole ARN in VEH- and PNA-treated mice. Results are presented as mean ± SEM. **, *P* < 0.01 as determined by a 2-tailed unpaired Student *t* test. Scale bars = 50 µm, scale bar in inset 10 µm.

 Nuclear progesterone receptor (PR)-ir was found predominantly in the dorsomedial and ventrolateral subregions of the ARN, with very few stained nuclei evident in the ventromedial regions where NPY neuron somata are present (Fig. 3A). PR-ir was less abundant and less intense in PNA-treated mice (Fig. 3B) compared with VEH-treated mice (Fig. 3A). The mean total number of GFP-ir NPY neurons was not different between VEH-treated and PNA-treated mice in the ARN as a whole (Fig. 3C), nor in any specific rostral to caudal zone (Table 1). Significantly fewer PR-ir cells were evident in PNA-treated mice through the ARN (336.2 ± 17.11 cells) compared with VEH-treated mice (414.5 ± 6.80; *P* = 0.0013; Fig. 3D). When the rARN, mARN, and cARN were compared separately, 2-way ANOVA indicated an effect of treatment on the number of PR-ir cells (F [1, 48] = 13.07, *P* = 0.0007; Table 1), and post hoc analysis indicated significantly fewer PR-ir cells specifically in the rARN of PNA mice (Table 1). The co-expression of PR within NPY^ARN^ neurons was almost entirely absent [0.38 ± 0.2% in VEH-treated mice, and 0.25 ± 0.1% of NPY neurons in PNA-treated mice co-expressing PR (Table 1)].

#### Estrogen receptor alpha.

**Figure 4.**
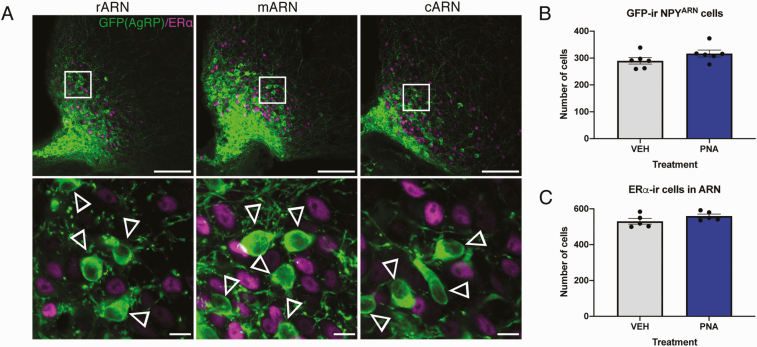
**ERα co-expression with NPY** ^**ARN**^  **neurons is absent in both VEH- and PNA-treated mice.** (A, top) Representative images composed from z-projections (6.36 µm thickness) collected throughout the ARN of a VEH-treated mouse showing ERα-ir (magenta) and τGFP reporter expression of NPY^ARN^ neurons (green). (A, bottom) single plane high magnification images taken within the highlighted area indicated in the top row. Individual single-labeled NPY neuron cell bodies are indicated by the empty arrowheads, while ERα-ir nuclei are shown in close proximity. (B) Mean number of GFP-expressing NPY neurons in VEH- (gray bars, n = 5) and PNA-treated (blue bars, n = 5) mice across the whole ARN. (C) Mean number of ERα-ir cells counted in VEH- and PNA-treated mice across the whole ARN. Results are presented as mean ± SEM. No significant differences were detected by 2-tailed unpaired Student *t* test. Scale bars = 100 µm (top), 10 µm (bottom).

 Images collected throughout the ARN displayed typical ventromedial localization of GFP-ir NPY neurons, whereas nuclear estrogen receptor alpha (ERα)-ir was scattered around the entire ARN (Fig. 4A, top). High magnification images (Fig. 4A, bottom) revealed that while ERα-ir nuclei lay in close proximity to NPY^ARN^ neuron cell bodies, they did not co-localize with NPY^ARN^ neurons. The mean total number of GFP-ir NPY neurons was not different between VEH-treated and PNA-treated mice in the ARN as a whole (Fig. 4B), nor in any specific rostral to caudal zone (Table 1). No difference in the expression of ERα was present between VEH- and PNA-treated mice (VEH 530.9 ± 15.99 cells vs PNA 559.7 ± 11.15 cells; Fig. 4C) within the ARN, nor in any particular rostral to caudal region (Table 1). The co-expression of ERα within NPY^ARN^ neurons was entirely absent in VEH-treated mice, and extremely limited in PNA-treated mice (0.15 ± 0.08%, Table 1).

## 3. Discussion

The present study assessed the impact of prenatal androgen excess, which models PCOS features, on the NPY^ARN^-to-GnRH neuron circuit. Using transgenic reporter expression specific to NPY^ARN^ neurons, we identified extensive NPY^ARN^ neuron projections to GnRH neurons, particularly to those in the rPOA and AHA. Confocal analysis of close appositions between NPY^ARN^ neurons fibers and GnRH neurons found no differences in the NPY^ARN^-to-GnRH neuron projection in PCOS-like PNA females. These findings suggest that this subset of GABA^ARN^ neurons are distinct to those that are remodeled by prenatal androgen excess [14, 16, 18]. Although NPY^ARN^ neurons were found to have virtually no co-expression with PR and ERα, irrespective of prenatal treatment, we did observe a greater proportion of NPY^ARN^ neurons co-expressing AR in PNA-treated mice, suggesting an upregulation of AR in NPY^ARN^ neurons in the PCOS-like condition. These findings indicate that although the NPY^ARN^ population are sensitive to androgens in adulthood, the NPY^ARN^-to-GnRH anatomical circuit is not obviously remodeled following prenatal androgen excess.

To dissect NPY/AgRP neurons and their full projections specifically originating from the ARN, AgRP-Cre mice were crossed with a line promoting Cre-dependant τGFP expression [42, 43]. This was an attractive approach, as AgRP and NPY are highly co-expressed in the ARN, and AgRP is exclusively expressed here [50-52]. This transgenic model is characterized as both highly specific and highly effective for identifying NPY^ARN^ neurons and their projections [35]. In addition, this study utilized a well-characterized model of PCOS suited to the study of the neuroendocrine pathology of PCOS [10]. This model exhibits the core reproductive abnormalities of PCOS, such as hyperandrogenaemia and anovulation, diminished ovarian hormone negative feedback, and LH hypersecretion [14, 20], without the associated metabolic syndrome [53, 54]. This allows for the characterization of circuit alterations that result from programmed androgen excess associated with reproductive function, without the confounding comorbid factors associated with obesity and hyperinsulinaemia present in other models of PCOS [10]. However, given the important role of NPY in energy balance, it would be of interest to investigate this circuit in models exhibiting the metabolic phenotype of PCOS.

The vast majority of GnRH neurons within the rPOA and AHA subpopulations received close contacts from NPY^ARN^ fibers, while only 5% of GnRH neurons in the MS subpopulation received NPY^ARN^ fiber contacts. In contrast, tract-tracing of the whole GABA^ARN^ population has found that approximately half of the MS GnRH neurons receive close appositions [14]. This suggests that GABA^ARN^ subpopulations have different projection patterns with respect to the specific GnRH neurons they innervate. This differential pattern of innervation to the GnRH neurons does not appear to have been previously reported, and supports an ongoing yet unproven hypothesis that GnRH neurons at different regions along their anatomical axis represent functional subpopulations. For example, c-Fos expression suggests that the activity and plasticity associated with LH surge generation is restricted to the rPOA subpopulation [55, 56] and receptor expression studies support the notion that the most rostral MS population may be differentially regulated for distinct functions [57, 58].

Putative innervation of GnRH neurons by NPY^ARN^ neurons was strikingly similar between PNA and VEH groups regardless of GnRH neural subpopulation or whether the soma or dendrite was examined. This analysis was refined further, where the density of contacts was assessed to normalize for variations in soma circumference between GnRH neurons, and by looking at 15-µm sections of the primary dendrite. In this instance, again, there were no differences between the VEH and PNA groups. The proximal GnRH neuron dendrite was the focus of this study, as GABAergic remodeling is restricted to this region [14, 16]. However, we cannot rule out changes at the more distal GnRH neuron dendron, the region known to be critical in driving pulsatile LH secretion [59]. In any case, the present finding stands in contrast to the plasticity that has been observed in the GABA^ARN^-to-GnRH neuron circuit, despite NPY^ARN^ neurons composing a large proportion of this circuit [14, 31]. This demonstrates that another, as yet undefined population of GABA^ARN^ neurons must be undergoing plastic reorganization as a result of PNA treatment, while NPY^ARN^ neurons are a distinct, unchanged population.

Increased GnRH neuronal activity in PCOS-like PNA mice [15, 19, 21] may reflect a modified balance in the excitatory and inhibitory afferent input that GnRH neurons receive. While GABAergic signaling to GnRH neurons is largely excitatory through GABA_A_ receptors [60], and selective optogenetic and chemogenetic activation of GABA^ARN^ inputs to GnRH neurons promotes LH secretion [17], NPY and AgRP likely promote inhibition of the GnRH neurons and LH release. NPY has been shown to act via the Y_1_ or Y_4_ receptors to inhibit or excite GnRH neural firing rate [38]; however NPY binds to Y_1_R at a far greater affinity than Y_4_R [61, 62], so the likely net effect is inhibitory. At the cellular level, AgRP has been shown to be stimulatory to a small population of GnRH neurons [38], but also to block the excitatory effects of melanocortin receptor agonists [63]. In ovariectomized monkeys, AgRP administration suppresses LH pulsatility [64]. In addition, optogenetic and chemogenetic activation of NPY^ARN^ neurons inhibits LH secretion and slows LH pulse frequency in castrated animals [35]. Therefore, the absence of enhanced input from the NPY^ARN^ subpopulation of GABA^ARN^ neurons to GnRH neurons in PNA mice aligns with their hyperactive hypothalamic-pituitary-gonadal axis state.

Androgens can modulate the development of the NPY^ARN^ neuron population. Male mice and rats have a larger population of NPY^ARN^ neurons compared with females [31, 65]. Likewise, female ewes treated with testosterone or DHT exhibit greater numbers of AgRP (thus, presumably NPY) neurons in the mARN [66], and prenatal androgen treatment in female ewes also produces greater AgRP fiber density in the POA and other hypothalamic areas [66]. NPY/AgRP neurons are implicated in neurodevelopmental processes; however, as shown here and reported previously [31], prenatally androgenized mice do not show any differences in NPY/AgRP cell numbers. The absence of NPY^ARN^ remodeling in the present study may suggest that this population is protected from androgen-driven plasticity in the developmental window that PNA treatment is applied. Although NPY/AgRP neurons are born on approximately embryonic day 12, significant development in this circuit occurs during the first 3 weeks of the postnatal period [67].

As PCOS-like PNA mice exhibit impaired steroid hormone negative feedback and reduced PR expression in the ARN [14, 20], we investigated the steroid hormone sensitivity within NPY^ARN^ neurons specifically. Immunohistochemistry confirmed reduced PR-expressing neurons within the ARN as reported previously [14]. Consistent again with this same study, the number of ERα-expressing cells in the ARN was unchanged. Despite evidence that NPY expression in the ARN varies over the estrous cycle of both the rat and mouse [68, 69], we found almost a complete absence of NPY^ARN^ co-expression with ERα or PR. Kim et al demonstrated a similarly low co-expression of ERα via immunohistochemistry in mice while also demonstrating that estradiol reduces NPY mRNA levels [70]. Estradiol regulation of NPY via classical receptors is therefore likely indirect, through peripheral pathways that then alter NPY expression. The lack of PR co-expression in NPY^ARN^ neurons found here is not surprising given the lack of ERα, and is in line with evidence in the ewe showing that NPY mRNA is not affected by progesterone administration, nor do NPY^ARN^ neurons possess PR [71]. These results indicate that the GABA^ARN^ neurons that lose PR expression in PNA mice [14] cannot consist of the NPY-expressing subpopulation.

In contrast to ERα and PR, AR was both co-expressed in NPY^ARN^ neurons and upregulated by PNA treatment. As circulating testosterone levels are elevated in PNA-treated mice [20], and AR expression in the brain appears to be positively autoregulated by androgens [72, 73], it is possible that hyperandrogenism in the PNA mouse is the driver of increased AR expression in NPY^ARN^ neurons. It remains to be determined whether elevated AR expression impacts neuroendocrine regulation in the PNA-treated mouse. Chronic DHT exposure from 3 weeks of age induces a range of metabolic effects including increased body mass and greater adiposity, and neuron-specific knockout of AR protects against DHT-induced metabolic and reproductive impairments [74]. Thus, excess androgens may positively regulate NPY^ARN^ neurons to increase orexigenic drive. However, prenatal androgen exposure that leads to postpubertal hyperandrogenism does not increase body weight and results in only very mild metabolic disturbances [53, 54], suggesting that AR signaling is not elevating NPY activity. Furthermore, as NPY/AgRP appear to inhibit GnRH neurons [38], and activated NPY^ARN^ neurons restrain LH secretion [35], elevated NPY^ARN^ activity would be at odds with the increased LH pulse frequency present in PNA-treated mice [20]. Therefore, further work is needed to dissect the actions of androgen signaling in NPY^ARN^ neurons and whether these actions play a role in the PCOS-like neuroendocrine impairments associated with prenatal androgen excess.

While NPY^ARN^ innervation to GnRH neurons appears unaltered, it remains possible that alterations to GnRH neuronal afferents are present; for example, NPY^ARN^ neurons project to and regulate kisspeptin neurons in the ARN [39]. Closer examination of these afferent circuits would give a more complete picture of whether PNA alters circuits in the pulse-generating networks of the hypothalamus. While more NPY^ARN^ neurons appear androgen-sensitive, this raises questions regarding the possible functional significance of this increase. As it stands, there is very little information in the mouse to suggest how androgens regulate NPY^ARN^ neurons. Future studies will be needed to determine whether androgens acting via AR in NPY^ARN^ neurons have any role in the interference of steroid hormone negative feedback within the PNA-treated mouse.

## Data Availability

The datasets generated during and/or analyzed during the current study are not publicly available but are available from the corresponding author on reasonable request.

## Abbreviations

AgRP: agouti-related peptide
AHA: anterior hypothalamic area
ANOVA: analysis of variance
AR: androgen receptor
ARN: arcuate nucleus
cARN: caudal arcuate nucleus
DHT: dihydrotestosterone
ERα: estrogen receptor alpha
GFP: green fluorescent protein
GnRH: gonadotropin-releasing hormone
ir: immunoreactive
LH: luteinizing hormone
mARN: middle arcuate nucleus
MS: medial septum
NPY: neuropeptide Y
PCOS: polycystic ovary syndrome
PNA: prenatally androgenized
PR: progesterone receptor
rARN: rostral arcuate nucleus
rPOA: rostral preoptic area
VEH: vehicle control

## Additional Information

***Disclosure Summary:*** The authors have nothing to disclose.
